# The antioxidant and Flavonoids contents of *Althaea officinalis* L. flowers based on their color

**Published:** 2012

**Authors:** Parisa Sadighara, Soraya Gharibi, Amir Moghadam Jafari, Golamreza Jahed Khaniki, Samira Salari

**Affiliations:** 1 *Department of Environmental Health, Food Safety Division, Faculty of Public Health, Tehran University of Medical Sciences, Tehran, I. R. Iran *; 2 *Academic Center of Education, Culture and Research (ACECR), Tehran, I. R. Iran *; 3 *Department of Toxicology, Ferdowsi University of Mashhad, Mashhad, I. R. Iran *; 4 *Department of Medical Mycology and Parasitology, School of Medicine, Kerman University of Medical Sciences, Kerman, I. R. Iran*

**Keywords:** Antioxidant Capability, Petals Color, Total Flavonoids

## Abstract

**Objective: **There has been a growing interest in finding plants with biological active ingredients for medicinal application.

**Materials and Methods:** Three colors of petals of *Althaea officinalis (A. officinalis)* flowers, i.e., pink, reddish pink, and white were examined for total antioxidant activity and ﬂavonoids content.

**Results: **The reddish pink flowers of *A. officinalis *have more antioxidant activity and the power of antioxidant activity was reddish pink > pink > white.

**Conclusion**: Findings suggest that the dark color can serve as an indicator of antioxidant content of the plant. Flavonoid content was highest in white flower thus this result indicated that flowers with light color can be considered for medicinal uses.

## Introduction

Plants have a significant role in maintaining human health and improving the quality of human life. The World Health Organization estimated that 80% of the people rely on traditional medicine (Craig, 1999 ▶). Out of the total flowering plants reported from the world, more than 50,000 are used for medicinal purposes (Teklehaymanot and Giday, 2007 ▶). In recent years, there has been a growing interest in finding plants with antioxidant activity for food and medicinal applications.

Reactive oxygen species (ROS) generation play a central role in pathogenesis of chronic diseases such as cancer, cardiovascular, rheumatoid arthritis, cataract, and others (Kourie, 1998 ▶). Therefore, dietary antioxidants should be consumed. Moreover, the antioxidant agents should be used in food industries for prevention of lipid peroxidation. Antioxidants products can be synthetic or natural. The use of synthetic antioxidants is restricted because of their toxicity, including carcinogenicity (Ashwini and Krishnamoorthy, 2011 ▶). Therefore, there has been growing interest in finding safe antioxidants.

*Althaea officinalis* *(A. officinalis) *is a medicinal plant consumed in case of lipemia, inflammation of nasal and oral cavities, gastric ulcer, platelet aggregation, cystitis, and irritating coughs (Sutovska et al., 2009 ▶; Hage-Sleiman et al., 2011 ▶). Its antioxidant activity has also been demonstrated. The extract of *A. officinalis *exhibited strong antioxidant activity in different antioxidant tests (Elmastas et al., 2004 ▶). Their antioxidant activity is accounted for approximately 69% of the activity of the reference compound alpha-tocopherol (Kardosova and Machova, 2006 ▶). This plant belongs to family Malvaceae, native to Europe and parts of Asia and is cultivated throughout the world. The flowers are terminal and axillary, with short peduncles, each bearing one, two, or three flowers. The petals are pale pink, reddish pink, and rarely, white, in color. 

The aim of this study was to compare the antioxidant activity and total ﬂavonoids content of *Althaea officinalis *L*.* flowers based on their color. 

## Materials and Methods

**Plant materials**

The flowers were collected in flowering season from a farm (Baghehfez farm, Tehran, Iran) and identified by Dr. Ziaei. Environmental conditions affect antioxidant capacity thus the flowers were collected from the same farm, in the same condition and time. The petals of flowers were separated, air dried in the shade and powdered. Half a gram of powder was soaked in 25 ml of ethanol-water (2:1) for 7 days. The extract was filtered and transferred to vials, and kept at 4 ˚C.

**Chemicals**

Neocuproine (Sigma Company, USA), FeCl_3_, KSCN, and AlCl_3_.6H_2_O (Merck Company, Germany) were used in the current study.

**Analysis of antioxidant activity (** **CUPRAC assay and ferric iron reducing assay)**

The CUPRAC assay was applied for analyzing total antioxidants capacity according to the previous method (Apak et al., 2007 ▶) with some modifications. The extraction was incubated with 10^-2^ M CuCl_2 _+7.5×10^-3^ Neocuproine + 1 M NH_4_Ac. The absorbance of the supernatant was measured at 450 nm. 

The ferric iron reducing assay was also applied for measuring total antioxidants. The extraction was incubated with 0.001 M FeCl_3 _and 0.06 M KSCN. The intensity of the colored species (Ferric thiocyanate) is measured using a spectrophotometer. Ferric thiocyanate complex was measured at 474 nm. A lower absorbance indicates an increased reducing power. The total antioxidant capacity was expressed by vitamin C equivalent antioxidant capacity. 

**Estimation of total ﬂavonoid compound**

Total flavonoid content was assayed according to previous methods (Qiu-Lin et al., 2006 ▶). Diluted extracts were mixed with reagent, AlCl_3_.6H_2_O 2% in methanol. Flavonoids could complex with trivalent aluminum ion. Then, the samples were incubated in room condition for 10 min. The absorbance of the samples was measured at 430 nm.

## Results

The results of CUPRAC assay and ferric iron reducing assay are shown in Table 1. The results were consistent in both CUPRAC and ferric iron reducing assays when the three different colored flowers were analyzed for measuring antioxidant activity. The antioxidant activities of herb extracts were expressed on the fresh weight basis as 1 ppm of vitamin C equivalent. Significant results were observed from these methods for reddish pinks. In both assays, reddish pink possessed the highest antioxidant activity compared with other extracts. 

**Table 1:** Level of antioxidant activity was expressed as equivalent vitamin C.

**The color of petals**	**The antioxidant activity** **(CUPRAC assay)**	**The antioxidant activity (ferric iron reducing assay)**
**White**	0.048±0.001	8.8±0.22
**Pink**	0.06±0.008	5.36±0.46
**Reddish pink**	0.11±0.006	4.74±0.14

Each value represents the mean ±SD per group. The values were significantly different in reddish pink extract compared with white and pink in CUPRAC assay (p=0.005).

**Table 2:** Results of flovonoid content

**The color of petals**	**The level of flavonoid**
**White **	48±3
**Pink **	32±1.5
**Reddish pink**	39.5±1.35

Each value represents the mean ±SD per group. The data were normalized to dry weight of plant

The data were normalized per 0.5 g of plant dry weight. High flavonoid content was observed in white flower followed by reddish pink and pink (Table 2). The level of flavonoid content was significantly different between white and other colors of the plant. 

## Discussion

In this study, the total antioxidant and total flavonoid content of three colors of* A. officinalis* were compared. To date, there are very few studies about the correlation between bioactivity of plants and their color. Environmental factors affect bioactivity of the plants. For instance, bilberry (*Vaccinium myrtillus* L.) preferably grows in shady places. When growing in the open, the color of upper leaves of plants turns red. This phenomenon provides protection against UV-B irradiation (Jaakola et al., 2009 ▶). In pervious studies, it was reported that the levels of antioxidant activity and content of total phenolics, total anthocyanins, total soluble solids, glucose, fructose, and acidity were higher in plants in the Mediterranean climate compared with those grown in the desert climate (Schwartz et al., 2009 ▶). The effect of air pollution on the antioxidant activity was confirmed in a study (Ghorbani et al., 2007 ▶). Moreover, the flavonoid synthesis is one of the general plant reactions to the stress (Mlodzinska, 2009 ▶). They are induced when plants are under low temperature or low nutrient state (Michalak, 2006 ▶). Therefore, we chose the flowers from a farm (Baghehfez farm).

Antioxidants are capable of deactivating free radical before the latter attack cells and biological targets (Atoui et al., 2005 ▶). Therefore, their activity will be critical for maintaining optimal protection. 

In the current study, the results suggest that the antioxidant activity has correlation with the intensity of plant color. Various methods have been developed in recent years to evaluate the total antioxidant activity of food. However, there is no accurate method that can be accounted for. Therefore, we evaluated total antioxidant activity of the flowers by two methods. In both methods, there was a possible link between the antioxidant activity and the color. Pigmented plants are outstanding sources for antioxidant compounds. Therefore, plants with deep color are excellent choices for antioxidant activity. Anthocyanins are powerful antioxidants and a major function of them is to provide color to most flowers, fruits, and vegetables. Anthocyanins contribute to the red, purple, and blue colors in plants. Therefore, there is a possible link between the anthocyanin level in the deep color of plant and their antioxidant capacity. 

In the current study, flavonoids content of pink and reddish flowers were less than white ones. Flavonoids are a group of polphenolic compounds. They are more than 4000 substance; mostly include flavonols, flavones, flavanone, anthocyanins, and catechins. The flavonoids exhibit a broad spectrum of pharmacological effects. In recent year, there have been drawing more attention to Alzheimer because of the considerable rate of this disease. Aluminum is neurotoxic and can induce many diseases such as Alzheimer and Parkinson's disease (Tian et al., 2006 ▶). Most flavonoids could bind to aluminum and decrease its content in the body (Qiu-Lin et al., 2006 ▶).The average human diet contains a considerable amount of flavonoids and the major dietary sources are fruits, vegetables, and different herbs (Sathishkumar et al., 2008 ▶). 

These components have broad pharmacological activity. They scavenge free radicals and can also improve the blood circulation, lower the blood pressure, anti-inflammatory, antibacterial, antiallergic, antiviral and estrogenic effects (Sathishkumar et al., 2008 ▶), inhibit lipid peroxidation, and prevent atherosclerosis (Giugliano, 2000 ▶). Flavonoids protect LDL from oxidation, inhibit platelet aggregation, have anti-inflammatory and antitumor agents (Craig, 1999 ▶) and create a UV protection filter (Mlodzinska, 2009 ▶). Moreover, some of them have antiproliferative and apoptotic effects on cancer cell lines (Intekhab and Aslam, 2009 ▶). In the present study, higher flavonoid content was found in white flowers. Sometimes, they have unexpected biological properties. For instance, white flowers are strong UV absorbers (Mlodzinska, 2009 ▶).

Collectively, the plants with more pigmentation have more biological effects in association with anti-oxidant potentials, and this fact should be considered by herbal manufactures and food industries in choosing antioxidant sources because our results showed dark colored flowers possess more antioxidant activity. Meanwhile, a significant variation was observed in amount of flavonoids in white flower compared with deep color plants. These data suggest flowers with light colors should be also considered for medical and health purposes. 
